# *μ*_3_-Oxido-hexa-*μ*_2_-pivalato-tris[(methanol-*κO*)cobalt(III)] chloride

**DOI:** 10.1107/S1600536809041907

**Published:** 2009-10-23

**Authors:** Yu Sun, Li-Li Zhu, Huai-Hong Zhang

**Affiliations:** a Ordered Matter Science Research Center, College of Chemistry and Chemical Engineering, Southeast University, Nanjing 210096, People’s Republic of China; b College of Pharmacy, Jiangsu University, Zhenjiang 212013, People’s Republic of China; c Department of Chemistry, Key Laboratory of Medicinal Chemistry for Natural Resource, Ministry of Education, Yunnan University, Kunming 650091, People’s Republic of China

## Abstract

The crystal structure of the title compound, [Co_3_(C_5_H_9_O_2_)_6_O(CH_4_O)_3_]Cl, consists of trinuclear Co^III^ complex cations and chloride anions. The Co^III^ cation has site symmetry *m*, and is coordinated by four oxygen atoms from four bridging pivalate groups, one central O anion and a methanol oxygen atom, forming a distorted octa­hedral geometry. The coordinated methanol mol­ecule is located on a crystallographic special position, the C and O atoms being located on the mirror plane. The central O anion lies in the crystallographic 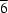 position, and acts as a *μ*_3_-O bridge, linking three equivalent Co^III^ cations and generating the oxo-centered trinuclear Co^III^ complex. The chloride anion, which acts as the counter-ion, is located on crystallographic 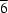 position. O—H⋯Cl hydrogen bonding between the Cl anion and hydroxyl group of the coordinated methanol mol­ecule links the mol­ecules into a supra­molecular network.

## Related literature

For oxido-centered triangular Co complexes, see: Aromì *et al.* (2003 ▶); Fursova *et al.* (2007 ▶). For related structures, see: Beattie *et al.* (1996 ▶).
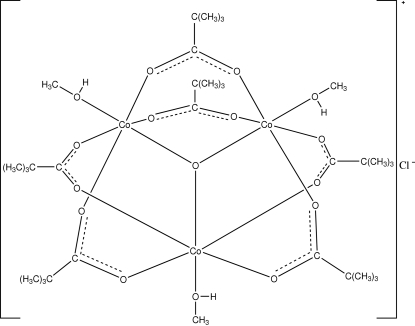


## Experimental

### 

#### Crystal data

[Co_3_(C_5_H_9_O_2_)_6_O(CH_4_O)_3_]Cl
*M* *_r_* = 931.10
Hexagonal, 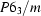

*a* = 10.4868 (15) Å
*c* = 22.794 (5) Å
*V* = 2170.9 (6) Å^3^
*Z* = 2
Mo *K*α radiation
μ = 1.26 mm^−1^
*T* = 293 K
0.16 × 0.14 × 0.11 mm

#### Data collection

Rigaku SCXmini 1K CCD area-detector diffractometer
Absorption correction: multi-scan (*CrystalClear*; Rigaku, 2005 ▶) *T*_min_ = 0.818, *T*_max_ = 0.871
9038 measured reflections
1317 independent reflections
1133 reflections with *I* > 2σ(*I*)
*R*_int_ = 0.046

#### Refinement

*R*[*F* ^2^ > 2σ(*F* ^2^)] = 0.037
*wR*(*F* ^2^) = 0.102
*S* = 1.11
1317 reflections
89 parameters
1 restraint
H atoms treated by a mixture of independent and constrained refinement
Δρ_max_ = 0.40 e Å^−3^
Δρ_min_ = −0.33 e Å^−3^

### 

Data collection: *CrystalClear* (Rigaku, 2005 ▶); cell refinement: *CrystalClear*; data reduction: *CrystalClear*; program(s) used to solve structure: *SHELXTL* (Sheldrick, 2008 ▶); program(s) used to refine structure: *SHELXTL*; molecular graphics: *SHELXTL*; software used to prepare material for publication: *SHELXTL*.

## Supplementary Material

Crystal structure: contains datablocks I, global. DOI: 10.1107/S1600536809041907/xu2614sup1.cif

Structure factors: contains datablocks I. DOI: 10.1107/S1600536809041907/xu2614Isup2.hkl

Additional supplementary materials:  crystallographic information; 3D view; checkCIF report

## Figures and Tables

**Table 1:** Hydrogen-bond geometry (Å, °)

*D*—H⋯*A*	*D*—H	H⋯*A*	*D*⋯*A*	*D*—H⋯*A*
O3—H3*D*⋯Cl1	0.90 (2)	2.17 (2)	3.070 (3)	175 (6)

**Fig. 1.**
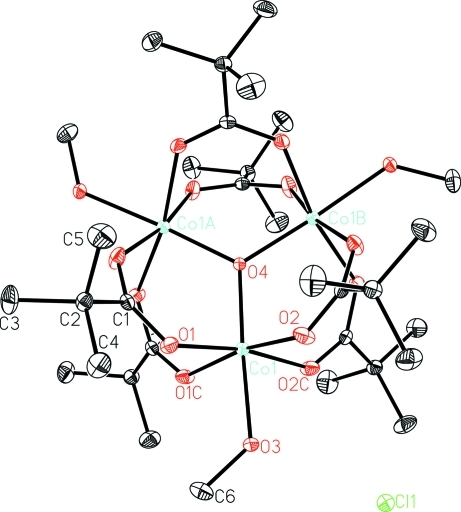
The molecular structure of the title molecule, with the atom-numbering scheme. Displacement ellipsoids are drawn at the 20% probability level. H atoms have been omitted for clarity [symmetry codes: (A) x, y, -z + 1/2; (B) -y + 1, x-y + 1, z; (C) -x + y, -x + 1, z].

**Fig. 2.**
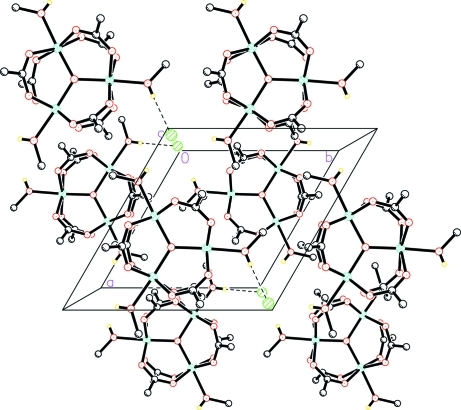
Crystal packing of the compound (I). The tertbutyl methyl groups and H atoms, except for those of the methanol ligands, have been omitted for clarity. Hydrogen bonds are shown as dashed lines.

## supplementary crystallographic information

### Comment

Oxo-centered triangular Co complexes, Co_3_(µ_3_-O), have been considered as effective models for studying M—*M* interactions in metal clusters (Aromì *et al.*, 2003; Fursova *et al.*,2007). We report here the synthesis and crystal structure of the title complex, (I). The complex is a typical oxo-centered carboxylate triangle, featuring exclusively, Co^III^ sites around a central µ_3_-oxide. Each edge of the triangle is further bridged by two pivalates with a terminal methanol ligand completing the coordination environment around each octahedral cobalt site (Fig. 1). The Co^III^ cation has site symmetry m. The central µ_3_-O lies in the -6 rotainversion axis. The coordinated methanol molecule is located on a crystallographic special position, the C and O atoms have site symmetry m. A similar oxo-centered cobalt(III) triangle has been reported for acetate (Beattie *et al.*, 1996). The O—H···Cl hydrogen bond between the Cl ion serving as a trifurcated acceptor and hydroxyl of the coordinated methanol molecule as donor link molecules into two-dimensional hydrogen-bonded networks (Fig. 2).

### Experimental

Hydrochloric acid (0.01 mmol) was added with constant stirring to a methanol solution (15 ml) containing Co(OOCC(CH_3_)_3_)_2_.4H_2_O (0.5 mmol), then filtered off. After a few days, red well shaped single crystals in the form of rectangular blocks deposited in the mother liquid. They were separated off, washed with cold methanol and dried in air at room temperature.

### Refinement

H atoms of the methyl groups were included in calculated positions and treated in the subsequent refinement as riding atoms, with C—H = 0.96 Å, *U*_iso_(H) = 1.2*U*_eq_(C). The hydride H3D was refined isotropically.

### Crystal data

**Table:**

[Co_3_(C_5_H_9_O_2_)_6_O(CH_4_O)_3_]Cl	*D*_x_ = 1.424 Mg m^−^^3^
*M**_r_* = 931.10	Mo *K*α radiation, λ = 0.71073 Å
Hexagonal, *P*6_3_/*m*	Cell parameters from 1832 reflections
Hall symbol: -P 6c	θ = 2.8–25.3°
*a* = 10.4868 (15) Å	µ = 1.26 mm^−^^1^
*c* = 22.794 (5) Å	*T* = 293 K
*V* = 2170.9 (6) Å^3^	Block, red
*Z* = 2	0.16 × 0.14 × 0.11 mm
*F*(000) = 980	

### Data collection

**Table:**

Rigaku SCXmini 1K CCD area-detector diffractometer	1317 independent reflections
Radiation source: fine-focus sealed tube	1133 reflections with *I* > 2σ(*I*)
graphite	*R*_int_ = 0.046
Detector resolution: 8.192 pixels mm^-1^	θ_max_ = 25.0°, θ_min_ = 1.8°
thin–slice ω scans	*h* = −12→10
Absorption correction: multi-scan (*CrystalClear*; Rigaku, 2005)	*k* = −12→12
*T*_min_ = 0.818, *T*_max_ = 0.871	*l* = −23→26
9038 measured reflections	

### Refinement

**Table:**

Refinement on *F*^2^	Primary atom site location: structure-invariant direct methods
Least-squares matrix: full	Secondary atom site location: difference Fourier map
*R*[*F*^2^ > 2σ(*F*^2^)] = 0.037	Hydrogen site location: inferred from neighbouring sites
*wR*(*F*^2^) = 0.102	H atoms treated by a mixture of independent and constrained refinement
*S* = 1.11	*w* = 1/[σ^2^(*F*_o_^2^) + (0.0515*P*)^2^ + 1.7379*P*] where *P* = (*F*_o_^2^ + 2*F*_c_^2^)/3
1317 reflections	(Δ/σ)_max_ < 0.001
89 parameters	Δρ_max_ = 0.40 e Å^−^^3^
1 restraint	Δρ_min_ = −0.33 e Å^−^^3^

### Special details

**Table:**

Geometry. All e.s.d.'s (except the e.s.d. in the dihedral angle between two l.s. planes) are estimated using the full covariance matrix. The cell e.s.d.'s are taken into account individually in the estimation of e.s.d.'s in distances, angles and torsion angles; correlations between e.s.d.'s in cell parameters are only used when they are defined by crystal symmetry. An approximate (isotropic) treatment of cell e.s.d.'s is used for estimating e.s.d.'s involving l.s. planes.
Refinement. Refinement of *F*^2^ against ALL reflections. The weighted *R*-factor *wR* and goodness of fit *S* are based on *F*^2^, conventional *R*-factors *R* are based on *F*, with *F* set to zero for negative *F*^2^. The threshold expression of *F*^2^ > σ(*F*^2^) is used only for calculating *R*-factors(gt) *etc*. and is not relevant to the choice of reflections for refinement. *R*-factors based on *F*^2^ are statistically about twice as large as those based on *F*, and *R*- factors based on ALL data will be even larger.

### Fractional atomic coordinates and isotropic or equivalent isotropic displacement parameters (Å^2^)

**Table:**

	*x*	*y*	*z*	*U*_iso_*/*U*_eq_	Occ. (<1)
Co1	0.51834 (5)	0.84488 (5)	0.2500	0.0226 (2)	
Cl1	1.0000	1.0000	0.2500	0.0400 (5)	
O4	0.3333	0.6667	0.2500	0.0196 (9)	
O3	0.7249 (3)	1.0326 (3)	0.2500	0.0325 (7)	
O2	0.6044 (2)	0.7791 (2)	0.18650 (10)	0.0401 (6)	
O1	0.4642 (2)	0.9451 (2)	0.18684 (9)	0.0343 (5)	
C1	0.3430 (3)	0.9136 (3)	0.16434 (12)	0.0249 (6)	
C2	0.3400 (3)	0.9828 (3)	0.10576 (13)	0.0303 (7)	
C3	0.2450 (4)	1.0559 (4)	0.11139 (17)	0.0530 (10)	
H3A	0.1473	0.9838	0.1234	0.079*	
H3B	0.2408	1.0966	0.0742	0.079*	
H3C	0.2876	1.1331	0.1401	0.079*	
C4	0.4953 (4)	1.0946 (4)	0.08584 (16)	0.0508 (9)	
H4B	0.5528	1.0470	0.0821	0.076*	
H4C	0.5402	1.1723	0.1143	0.076*	
H4D	0.4908	1.1351	0.0486	0.076*	
C6	0.7597 (6)	1.1820 (5)	0.2500	0.0530 (14)	
H6A	0.8647	1.2450	0.2500	0.080*	
H6B	0.7190	1.2010	0.2844	0.080*	0.50
H6C	0.7190	1.2010	0.2156	0.080*	0.50
C5	0.2695 (5)	0.8587 (5)	0.06128 (16)	0.0605 (11)	
H5A	0.3284	0.8126	0.0574	0.091*	
H5B	0.2630	0.8977	0.0240	0.091*	
H5C	0.1726	0.7873	0.0743	0.091*	
H3D	0.801 (5)	1.016 (7)	0.2500	0.08 (2)*	

### Atomic displacement parameters (Å^2^)

**Table:**

	*U*^11^	*U*^22^	*U*^33^	*U*^12^	*U*^13^	*U*^23^
Co1	0.0220 (3)	0.0214 (3)	0.0235 (3)	0.0101 (2)	0.000	0.000
Cl1	0.0312 (6)	0.0312 (6)	0.0575 (13)	0.0156 (3)	0.000	0.000
O4	0.0184 (13)	0.0184 (13)	0.022 (2)	0.0092 (7)	0.000	0.000
O3	0.0204 (14)	0.0222 (14)	0.0514 (19)	0.0081 (12)	0.000	0.000
O2	0.0346 (12)	0.0285 (11)	0.0463 (14)	0.0076 (9)	0.0183 (10)	−0.0086 (10)
O1	0.0280 (11)	0.0297 (11)	0.0382 (13)	0.0091 (9)	−0.0056 (9)	0.0128 (9)
C1	0.0303 (15)	0.0219 (13)	0.0251 (15)	0.0150 (11)	−0.0001 (12)	0.0010 (11)
C2	0.0311 (16)	0.0334 (15)	0.0274 (16)	0.0170 (13)	0.0008 (12)	0.0073 (12)
C3	0.061 (2)	0.068 (2)	0.049 (2)	0.047 (2)	0.0104 (18)	0.0244 (19)
C4	0.045 (2)	0.057 (2)	0.046 (2)	0.0223 (17)	0.0121 (17)	0.0272 (18)
C6	0.052 (3)	0.028 (2)	0.067 (4)	0.011 (2)	0.000	0.000
C5	0.081 (3)	0.064 (2)	0.032 (2)	0.032 (2)	−0.0127 (19)	−0.0090 (18)

### Geometric parameters (Å, °)

**Table:**

Co1—O4	1.9055 (5)	C2—C5	1.519 (5)
Co1—O2^i^	2.002 (2)	C2—C4	1.524 (4)
Co1—O2	2.002 (2)	C2—C3	1.537 (4)
Co1—O1^i^	2.0244 (19)	C3—H3A	0.9600
Co1—O1	2.0244 (19)	C3—H3B	0.9600
Co1—O3	2.074 (3)	C3—H3C	0.9600
Cl1—Cl1	0.0000	C4—H4B	0.9600
O4—Co1^ii^	1.9055 (6)	C4—H4C	0.9600
O4—Co1^iii^	1.9055 (6)	C4—H4D	0.9600
O3—C6	1.420 (5)	C6—H6A	0.9600
O3—H3D	0.90 (2)	C6—H6B	0.9600
O2—C1^iii^	1.252 (3)	C6—H6C	0.9600
O1—C1	1.251 (3)	C5—H5A	0.9600
C1—O2^ii^	1.252 (3)	C5—H5B	0.9600
C1—C2	1.528 (4)	C5—H5C	0.9600
			
O4—Co1—O2^i^	94.33 (6)	C4—C2—C1	111.0 (2)
O4—Co1—O2	94.33 (6)	C5—C2—C3	108.9 (3)
O2^i^—Co1—O2	92.60 (15)	C4—C2—C3	110.5 (3)
O4—Co1—O1^i^	95.56 (6)	C1—C2—C3	109.7 (3)
O2^i^—Co1—O1^i^	87.52 (10)	C2—C3—H3A	109.5
O2—Co1—O1^i^	170.07 (9)	C2—C3—H3B	109.5
O4—Co1—O1	95.56 (6)	H3A—C3—H3B	109.5
O2^i^—Co1—O1	170.07 (9)	C2—C3—H3C	109.5
O2—Co1—O1	87.52 (10)	H3A—C3—H3C	109.5
O1^i^—Co1—O1	90.66 (13)	H3B—C3—H3C	109.5
O4—Co1—O3	177.12 (8)	C2—C4—H4B	109.5
O2^i^—Co1—O3	83.69 (8)	C2—C4—H4C	109.5
O2—Co1—O3	83.69 (8)	H4B—C4—H4C	109.5
O1^i^—Co1—O3	86.46 (8)	C2—C4—H4D	109.5
O1—Co1—O3	86.46 (8)	H4B—C4—H4D	109.5
Co1^ii^—O4—Co1	120.0	H4C—C4—H4D	109.5
Co1^ii^—O4—Co1^iii^	120.0	O3—C6—H6A	109.5
Co1—O4—Co1^iii^	120.0	O3—C6—H6B	109.5
C6—O3—Co1	128.1 (3)	H6A—C6—H6B	109.5
C6—O3—H3D	117 (4)	O3—C6—H6C	109.5
Co1—O3—H3D	115 (4)	H6A—C6—H6C	109.5
C1^iii^—O2—Co1	134.27 (18)	H6B—C6—H6C	109.5
C1—O1—Co1	131.72 (18)	C2—C5—H5A	109.5
O1—C1—O2^ii^	123.9 (3)	C2—C5—H5B	109.5
O1—C1—C2	119.5 (2)	H5A—C5—H5B	109.5
O2^ii^—C1—C2	116.6 (2)	C2—C5—H5C	109.5
C5—C2—C4	109.6 (3)	H5A—C5—H5C	109.5
C5—C2—C1	107.0 (3)	H5B—C5—H5C	109.5
			
O2^i^—Co1—O4—Co1^ii^	133.53 (7)	O1—Co1—O2—C1^iii^	−111.7 (3)
O2—Co1—O4—Co1^ii^	−133.53 (7)	O3—Co1—O2—C1^iii^	161.6 (3)
O1^i^—Co1—O4—Co1^ii^	45.60 (6)	O4—Co1—O1—C1	12.6 (3)
O1—Co1—O4—Co1^ii^	−45.60 (6)	O2—Co1—O1—C1	106.7 (3)
O2^i^—Co1—O4—Co1^iii^	−46.47 (7)	O1^i^—Co1—O1—C1	−83.1 (3)
O2—Co1—O4—Co1^iii^	46.47 (7)	O3—Co1—O1—C1	−169.5 (3)
O1^i^—Co1—O4—Co1^iii^	−134.40 (6)	Co1—O1—C1—O2^ii^	16.6 (4)
O1—Co1—O4—Co1^iii^	134.40 (6)	Co1—O1—C1—C2	−162.2 (2)
O2^i^—Co1—O3—C6	−133.34 (7)	O1—C1—C2—C5	114.9 (3)
O2—Co1—O3—C6	133.34 (7)	O2^ii^—C1—C2—C5	−64.0 (3)
O1^i^—Co1—O3—C6	−45.44 (6)	O1—C1—C2—C4	−4.6 (4)
O1—Co1—O3—C6	45.44 (6)	O2^ii^—C1—C2—C4	176.5 (3)
O4—Co1—O2—C1^iii^	−16.3 (3)	O1—C1—C2—C3	−127.0 (3)
O2^i^—Co1—O2—C1^iii^	78.2 (3)	O2^ii^—C1—C2—C3	54.0 (4)

Symmetry codes: (i) *x*, *y*, −*z*+1/2;  (ii) −*y*+1, *x*−*y*+1, *z*; (iii) −*x*+*y*, −*x*+1, *z*.

### Hydrogen-bond geometry (Å, °)

**Table:**

*D*—H···*A*	*D*—H	H···*A*	*D*···*A*	*D*—H···*A*
O3—H3D···Cl1	0.90 (2)	2.17 (2)	3.070 (3)	175 (6)
